# Role of the Health Belief Model in the Management of Hypertension: A Systematic Review

**DOI:** 10.7759/cureus.94139

**Published:** 2025-10-08

**Authors:** Mohammad Tanvir Islam, Shohael Mahmud Arafat, Arna Chowdhury, Keertika Orchi, Shahana Sultana, Tanjela Bushra, Md Redwanul Islam, Khandakar Fatema, Md Maruf Haque Khan, M Atiqul Haque

**Affiliations:** 1 Department of Internal Medicine, Bangabandhu Sheikh Mujib Medical University, Dhaka, BGD; 2 Department of Public Health and Informatics, Bangabandhu Sheikh Mujib Medical University, Dhaka, BGD

**Keywords:** bp control, health belief model, hypertension, medication adherence, nonpharmacological therapy, self-management

## Abstract

Theory-driven behavioral models such as the health belief model (HBM) offer predictive and influential insights into hypertension management. This study aims to explore the role of the health belief model in hypertension management, with a focus on blood pressure control, medication adherence, and self-management.

This review included English full-text quantitative studies on HBM and hypertension management in low- and middle-income countries (LMICs), excluding qualitative, mixed methods, protocols, and nonoriginal data. The review was registered in PROSPERO (PROSPERO 2023 CRD42023467247) and conducted in accordance with the Preferred Reporting Items for Systematic Reviews and Meta-Analyses (PRISMA) guidelines. A comprehensive search was carried out across six electronic databases, PubMed, APA PsycINFO, CINAHL, Scopus, Embase, and the Cochrane Library, between September 26 and October 2, 2023, to identify relevant published studies. The risk of bias was assessed via the RoB-2 (The Risk of Bias 2) and JBI (Joanna Briggs Institute) Critical Appraisal Tools. Data were extracted into an Excel data sheet (Microsoft Corporation, Redmond, Washington) for result synthesis and tabulation.

An initial total of 1,064 articles were identified for review. Following the removal of duplicates and a full-text assessment, 24 articles with a sample size of 6,106 met the inclusion criteria. The application of interventions based on the HBM constructs was associated with reduced blood pressure, improved medication adherence, and self-management. Most studies have shown that perceived susceptibility, severity, and self-efficacy are positively associated with BP reduction, whereas perceived barriers have a negative impact on adherence. Perceived susceptibility and self-efficacy are also frequently linked to better self-management.

The HBM has the potential to predict health behaviors among individuals with hypertension. Interventions based on the HBM offer potential for effective hypertension control.

## Introduction and background

Hypertension is a global threat, leading to significant cardiovascular morbidity and affecting one in every three people [1]. Controlling hypertension can significantly reduce the risk of cardiovascular events, stroke, renal and retinal complications, and all-cause mortality [2,3]. In the past few decades, while high-income countries have experienced a decline in hypertension rates, low- and lower-middle-income countries continue to face a high prevalence [4,5]. In low- and middle-income countries (LMICs), undetected hypertension has become a pressing concern, with a prevalence ranging from 15% to 55%, and only 10% of diagnosed cases achieving the target blood pressure (BP) reduction [6-11].

Medication adherence, which refers to the extent to which a patient follows prescribed medications, dietary guidelines, and lifestyle changes, is a critical factor in managing hypertension [12]. Suboptimal adherence to hypertension treatment presents a significant challenge in low- and middle-income countries [13]. In addition, individuals with hypertension in these regions often exhibit poor self-management of their condition [14]. Self-management is a dynamic process involving daily activities that help individuals monitor and manage disease-related events and adopt healthy lifestyle behaviors [15]. Research has demonstrated that self-management plays a significant role in improving both medication adherence and blood pressure reduction [16].

Understanding health behaviors is key to promoting effective long-term management at both individual and societal levels [17]. The incorporation of theoretical models in interventions to control chronic illnesses such as diabetes and hypertension has proven to be more effective [18]. Conner and Norman described health behavior as an action aimed at preventing or detecting disease or improving overall well-being [19]. Several theories explain health-promoting behaviors, including the Health Belief Model (HBM), Transtheoretical Model (TTM), Social Cognitive Theory, Self-Regulation Theory, and Social Ecological Theory, with HBM being the most widely applied [20].

The HBM was developed by Rosenstock and his team in the 1950s and has since become one of the most recognized behavioral theories in the following decades [21-24]. The model consists of six components: perceived susceptibility, perceived severity, perceived benefit, perceived barrier, cues to action, and self-efficacy. The initial model started with four components, and later, “self-efficacy” and “cues to action” were incorporated as integral parts of the model [25]. The model aims to explain how an individual's beliefs impact their involvement in health-related behaviors. According to the HBM, if someone feels at risk (perceived susceptibility), such as having a family history of stroke, they might be more motivated to control their hypertension. Action is further encouraged by acknowledging the grave implications of uncontrolled blood pressure (perceived severity), such as heart attack or renal failure. Although obstacles such as expense, adverse effects, or lifestyle modifications (perceived barriers) may make adherence difficult, motivation increases when one believes that preventive measures such as taking medication, reducing salt intake, and exercising can minimize these risks (perceived benefit). Self-efficacy, or the belief that one can follow treatment independently, helps maintain long-term control. Cues to action, including a doctor’s advice or witnessing a relative’s illness, might also trigger behavior change [26]. In addition to these core components, sociodemographic factors such as age, sex, and health literacy can influence a person’s perception of illness [27].

Previous reviews of the HBM have explored its role in preventive measures such as screening for different illnesses [28-30]. Reviews and meta-analyses have examined the effectiveness of the HBM in predicting health behaviors [23,24,31,32]. A systematic review also assessed its impact on medication adherence [33]. None of these studies focused on investigating the role of the HBM or HBM-based interventions in the management of hypertension. Since its introduction, numerous studies have highlighted the impact of interventions based on the HBM in managing hypertension [34-36]. However, review articles summarizing these effects and identifying the strengths and limitations of using the HBM in hypertension management are lacking. Given the high incidence and poor control of hypertension in resource-limited countries, it is crucial to identify an effective model that can address this issue.

Therefore, our research question was whether the HBM has any role in the management of hypertension, particularly among individuals from LMICs. The primary objective of this systematic review was to explore the role of the HBM in controlling hypertension in LMICs and, secondarily, to assess its impact on treatment adherence and self-management, which are key factors in determining treatment success.

## Review

Methods

We reviewed studies providing information on the associations between HBM constructs and hypertension in the adult population. A protocol (PROSPERO 2023 CRD42023467247) was registered for this systematic review and adhered to the Preferred Reporting Items for Systematic Reviews and Meta-Analyses (PRISMA) guidelines.

Eligibility criteria

Inclusion and Exclusion Criteria

The inclusion criteria for this systematic review include the following: (1) articles providing information on the association of the HBM with hypertension management; (2) the study population consisted of hypertensive adult individuals in LMICs; (3) full-text articles published in English; and (4) primary articles with quantitative study designs, including cross-sectional, longitudinal, and intervention studies, as well as relevant gray literature (non-peer-reviewed or in preprint form). Articles were excluded if they (1) did not fulfill the objectives of the review; (2) included a study population below 18 years of age; (3) did not provide original data (e.g., research reports, reviews, editorials, dissertations); or (4) were protocol studies.

Intervention and Outcome

This systematic review examined experimental studies that used interventions based on different constructs of the HBM, as well as other quantitative studies that did not utilize a specific intervention but used the HBM as a framework, for questionnaire formulation, or for other methodological purposes. Comparators for the experimental studies included either no intervention, standard care, or alternative approaches.

The primary outcome of this study was BP reduction, and the secondary outcomes were changes in medication adherence scores and self-management scores.

Information Sources

This review systematically searched six electronic databases: PubMed, American Psychological Association (APA) PsycINFO, CINAHL, Scopus, Embase, and the Cochrane Library. The search was conducted between September 26 and October 2, 2023, covering relevant published articles from the date of database inception (the earliest identified article was published in 1974).

Search Strategy

The search strings were developed around two key concepts: hypertension and the health belief model. To ensure that all relevant documents were included, the database search began with the development of initial search strings for PubMed, which were as follows: "hypertension"[mh] OR "high blood pressur*"[all] OR "blood pressur*"[all] OR "BP"[all] OR "elevated blood pressur*"[all] OR "raised blood pressur*"[all] AND "Health belief model"[mh] OR "Health belief model"[all]. Using these initial strings, we then created the final search strings, which were applied to each database. We used truncations, wildcards, Boolean operators, and Medical Subject Headings (MeSH) to identify relevant documents during the search process.

Finally, we searched reference lists and other articles in the gray literature to ensure that the comprehensive search included studies meeting the inclusion criteria. During the search process, we used truncations, wildcards, Boolean operators, and Medical Subject Headings to find relevant documents.

Data extraction and management

Selection Process and Data Collection Process

The titles from all databases were exported into Mendeley reference management software, version 1.19.8 (Mendeley Ltd., London, United Kingdom), for compilation, and duplicates were removed. The compiled list was then imported into Rayyan (Rayyan Systems Inc., Cambridge, Massachusetts), a web-based application for screening articles in systematic reviews. Three independent reviewers (M.R.I., K.O., and A.C.) participated in all stages of the screening process within Rayyan, followed by a full-text assessment for relevance and quality. In cases of disagreement during the screening process, the first author (M.T.I.) resolved the issues.

After identifying the studies for this review, the authors held a roundtable workshop to ensure accurate extraction of relevant data and variables. A pilot test was conducted by the independent reviewers, and refinements were made based on feedback. All authors reviewed selected full-text articles and decided which variables should be extracted for this review. Data extraction was subsequently performed and documented in an Excel spreadsheet (Microsoft Corporation, Redmond, Washington) by two authors (A.C. and K.O.). The first author (M.T.I.) reviewed the extracted data, and any differences were resolved by consensus.

Data Items

The following information was extracted and summarized from the selected articles: author(s), year of publication, article title, journal name, study design, and study objectives. The extracted data also included study location, sample size, study population, age group, data collection methodology, intervention, and outcomes.

A comprehensive evaluation of the selected articles was carried out, emphasizing the application of the HBM. The constructs of the HBM were identified across all studies. Data were also extracted to examine the role of the six components of the HBM in relation to hypertension, the frequency with which these constructs were explained, and the concepts they represented. In addition to identifying the use of the HBM, the analysis explored how the different components of the model were applied to lower blood pressure, promote self-care behaviors, and ensure drug adherence.

Data Synthesis

This review primarily aimed to narratively synthesize the studies, their participants, the application of the HBM, and the outcomes associated with hypertension management. Studies were categorized into three domains: BP reduction, medication adherence, and self-management. Outcome measures were presented in tabulated form, illustrating the associations of different constructs of the HBM with these domains as statistically significant or clinically relevant. Changes in blood pressure were evaluated by comparing mean values before and after the intervention. Changes in mean scores were used to evaluate medication adherence and self-management outcomes. “Missing constructs” and “Not mentioned” labels were used to denote unreported and unutilized HBM components.

Quality Assessment and Risk of Bias

**Figure 1:**
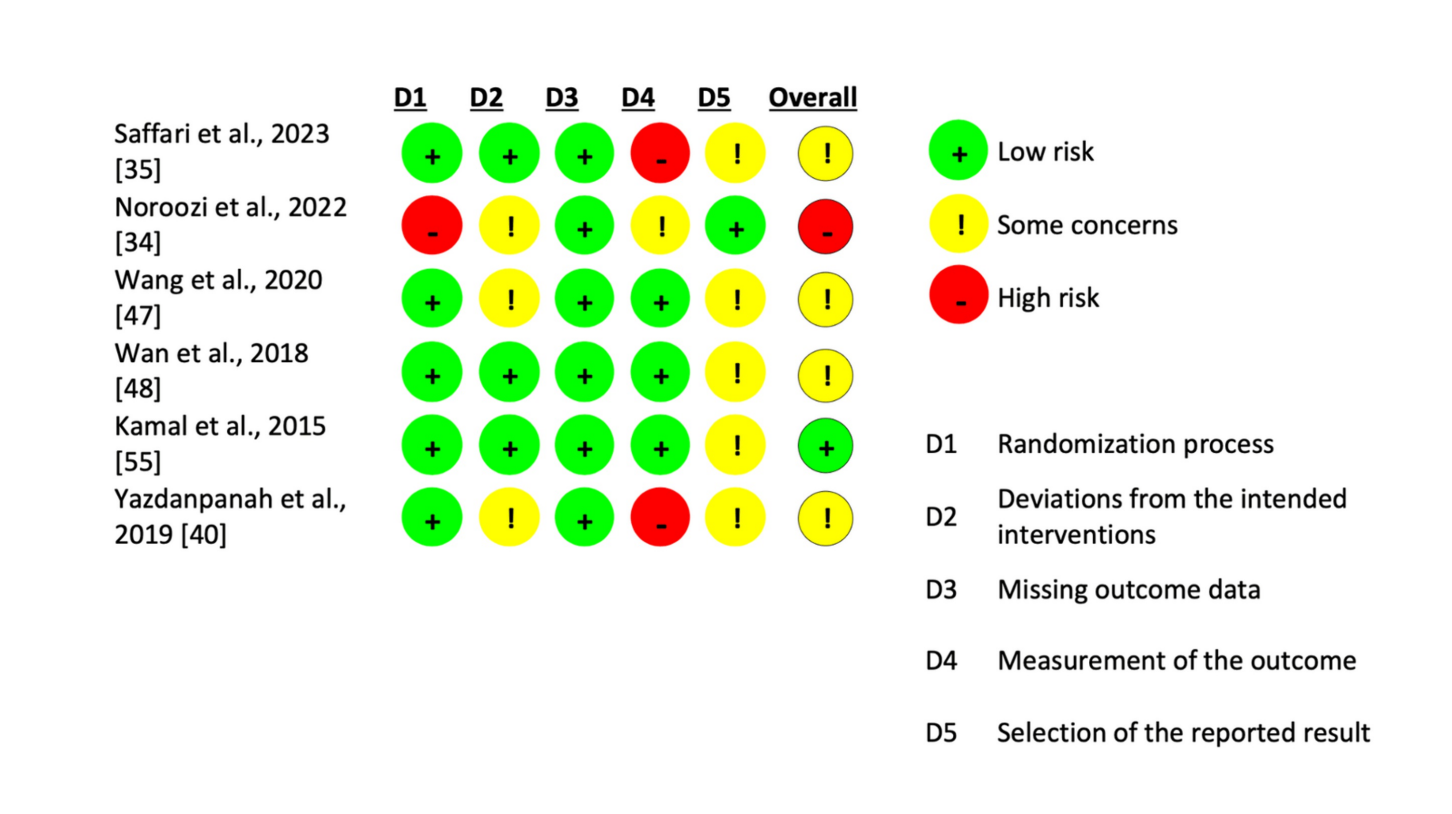
Risk of Bias evaluation for RCT studies (RoB-2). RCT: randomized controlled trial.

The risk of bias was evaluated using the revised Cochrane risk of bias tool (RoB-2) for RCTs (n = 6). The remaining 18 studies were assessed using the Joanna Briggs Institute (JBI) Critical Appraisal Tools with individual checklists for cross-sectional, cohort, and quasi-experimental studies. Scoring of the JBI tool assessments was performed following the approach outlined by Manosroi et al. [37]. Two RCTs were found to have a low risk, three showed some concerns, and one had a high risk of bias (Figure 1). Among the 18 studies included, 12 had a moderate risk of bias, whereas six had a low risk of bias. Detailed information on the JBI checklists is available in the Appendices. Two authors (A.C. and K.O.) conducted the quality assessment, and the first author (M.T.I.) reviewed it.

Results

Search Results

**Figure 2:**
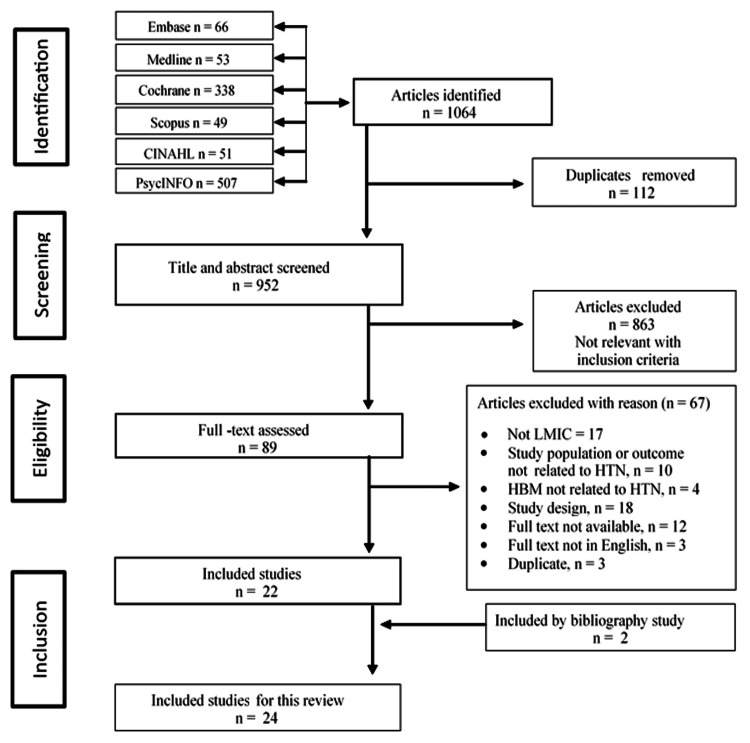
PRISMA flow diagram of the flow of the studies through each phase of the screening process. PRISMA: Preferred Reporting Items for Systematic Reviews and Meta-Analyses.

We initially identified 1,064 articles for review. After eliminating 112 duplicates and conducting a preliminary screening of titles and abstracts, the number of articles was reduced to 89. A full-text screening of these articles was then performed, resulting in the inclusion of 22. Sixty-six articles were excluded for various reasons. Additionally, two articles were added through a review of bibliographies, bringing the total number of included articles to 24 (Figure 2).

Characteristics of Included Studies

Geographically, 11 of these studies were from Iran [34,35,38-46], six from China [36,47-51], two from Egypt [52,53], and one each from India [54], Pakistan [55], Indonesia [56], Ghana [57], and Brazil [58]. The studies covered a 20-year period, with 13 published in the 2010s and 11 between 2020 and 2023. The study populations comprised adults aged 18 years and older. The total combined sample size was 6,106, with individual study samples ranging from 58 to 745 participants. Of the 24 studies, six were RCTs, four were quasi-experimental, 12 were cross-sectional, and the remaining two were pre- and post-intervention and nested cohort studies.

HBM Constructs Presented in the Studies

As the HBM has evolved over time, the number of components utilized in the studies has varied [25]. Not all studies incorporated the full set of HBM constructs, and the authors did not provide explanations for their exclusion. Specifically, 14 studies utilized all six constructs of the Health Belief Model, eight incorporated five constructs, and the remaining studies included only four components in their analyses. The most frequently missing constructs were “cues to action” and “self-efficacy,” followed by “perceived susceptibility” and “perceived severity.”

Relationship of constructs of HBM with different domains

Blood Pressure Reduction

This review examined the association of different constructs of the HBM and interventions based on them in the reduction of BP (Table 1). Among the nine studies reviewed, five were randomized controlled trials [34,35,40,47,48,55], two were quasi-experimental [38,45], and one each was a pre- and post-intervention and nested cohort study [36,52]. All studies were conducted in different parts of Asia and Africa. In addition to the HBM, social cognitive theory was also applied in one study [55]. The components of the interventions varied, including comprehensive reminder systems, workshop sessions, health counseling, and telephone follow-up. The duration of the interventions ranged from two weeks to six months, reflecting a diverse range of program intensities and timelines. The constructs that exhibited a positive association with BP reduction were perceived susceptibility, perceived severity, and self-efficacy.

**Table 1:** Association of interventions based on different constructs of HBM in BP reduction CRS-HBM: comprehensive reminder system–HBM; Study design: RCT: randomized controlled trial, QES: quasi-experimental study; +: positively significant; –: negatively significant; MC: missing construct; N/A: not applicable; NM: association not mentioned; NS: not significant.

Reference	Country and Sample Size	Study Design	Association With Different Constructs of HBM						Intervention	Effect of Intervention on BP	
			Perceived Susceptibility	Perceived Severity	Perceived Benefit	Perceived Barrier	Cues to Action	Self-Efficacy		Pre-intervention	Post-intervention
Saffari, 2023 [35]	Iran, 120	RCT	+	+	+	-	+	+	HBM-based education program; Over 6 weeks	Mean ± SD of Intervention group – SBP: 145.70 ± 10.80 mmHg, DBP: 88.80 ± 7.30 mmHg, and Control group – SBP: 144.20 ± 9.90 mmHg, DBP: 88.00 ± 7.40 mmHg	Mean ± SD of Intervention group – SBP: 139.40 ± 8.40 mmHg, DBP: 85.90 ± 6.40 mmHg; Control group – SBP: 145.20 ± 10.00 mmHg; DBP: 87.70 ± 7.0 mmHg; ANCOVA test (p value) – SBP: 97.3 (< 0.001), DBP: 22.4 (<0.001)
Noroozi, 2022 [34]	Iran, 200	RCT	+	+	+	-	MC	+	HBM-based education program; 8 workshop sessions in 8 weeks on blood pressure	Mean ± SD of Intervention group – SBP: 136.15 ± 13.74 mmHg, DBP: 81.46 ± 8.95 mmHg, and Control group – SBP: 136.16 ± 16.78 mmHg, DBP: 81.20 ± 10.06 mmHg	Mean ± SD of Intervention group – SBP: 133.86 ± 14.84 mmHg, DBP: 80.60 ± 8.29 mmHg; and Control group – SBP: 136.10 ± 16.64 mmHg, DBP: 81.25 ± 10.05 mmHg Mean difference in intervention group – SBP: 2.29 mmHg, DBP:1.04 mmHg; in control group – SBP:0.90 mmHg, DBP:0.75 mmHg; In intervention group – SBP (p value = 0.02) DBP (p value = 0.03)
Afshari, 2022 [45]	Iran, 100	QES	+	+	NS	NS	NS	+	HBM-based education program, 3 training sessions in 2 weeks, each lasting 1 hour		Mean ± SD of Intervention groups: 151.99 ± 1.98 mmHg; Control group – 152.94 ± 2.53 mmHg. No significant difference between the intervention vs. control groups (p>0.05)
Wang, 2020 [47]	China, 174	RCT	NM	NM	NM	NM	MC	NM	Intervention group – Usual care and (CRS-HBM) for 6 months, including: 1. Health belief education; 2. The HBM Calendar Handbook; 3. Weekly short message services; 4. Telephone follow-up interviews. Control: usual care	Mean ± SD of Intervention group – SBP: 142.91 ± 14.05 mmHg, DBP: 80.33 ± 9.12 mmHg; Control group – SBP: 138.65 ± 18.36 mmHg, DBP: 83.32 ± 13.86 mmHg	Mean ± SD of Intervention group – SBP: 132.14 ± 10.67 mmHg, DBP: 79.57 ± 7.41 mmHg; Control group – SBP: 137.37 ± 13.73 mmHg, DBP: 83.07 ± 9.70 mmHg; SBP (p value < 0.05) DBP (p value = 0.927)
Saffari, 2020 [38]	Iran, 58	QES	+	+	+	-	NS	+	HBM-based education program; 5 educational sessions	Mean ± SD: SBP: 132.3±26.8 mmHg DBP: 85.1±19.7 mmHg (p value = 0.163)	Mean ± SD of Intervention group – SBP: 126.2±24.9 mmHg; Control group – DBP: 83.5±18.8 mmHg SBP (p value = 0.063) DBP (p value = 0.235), Not significant
Zhang, 2020 [36]	China, 174	Nested Cohort	NM	NM	NM	NM	MC	NM	(CRS-HBM) for 6 months, including: 1. Health belief education; 2. The HBM Calendar Handbook; 3. Weekly short message services; 4. Telephone follow-up interviews	Mean ± SD of Intervention group – BP control: 41 ± 53.95 mmHg; Control group – BP control: 38 ± 50.67 mmHg (p value = 0.163)	Mean ± SD of Intervention group – BP control: 64 ± 84.2 mmHg; Control group – BP control: 37 ± 49.33 mmHg (p value < .001); On BP control; Direct positive effect (β = .356, p < .001); Indirect positive effects (β = .183, p = .009)
Wan, 2018 [48]	China, 174	RCT	NM	NM	NM	NM	NM	NM	Intervention group – Usual stroke education and (CRS-HBM) for 6 months, including: 1. Health belief education; 2. The HBM Calendar Handbook; 3. Weekly short message services; 4. Telephone follow-up interviews. Control group – usual stroke education	Mean ± SD of Intervention group – SBP: 143.21 ± 13.96 mmHg, DBP: 80.53 ± 9.02 mmHg; Control group – SBP: 138.96 ± 18.37 mmHg, DBP: 79.40 ± 11.00 mmHg	Mean difference in intervention group – SBP: -9.86 mmHg, DBP: -0.59 mmHg; in control group – SBP: -1.38 mmHg, DBP:+3.10 mmHg; From baseline to follow-up: Intervention group – SBP (p value<0.001), DBP (p value=0.583); Control group – SBP (p value=0.558), DBP (p value=0.038)
Allah and Khalil, 2016 [52]	Egypt, 186	Pre-post-intervention study	+	+	+	-	+	+	Intervention group – HBM-based education program. Control group – Traditional health education on hypertension and compliance to medication and lifestyle regimen	Mean ± SD: SBP: 169.3±19.4 mmHg, DBP: 97.6±7.3 mmHg (p value <0.001)	Mean ± SD of Intervention group – SBP: 150.2±14.5 mmHg; Control group – DBP: 87.1±6.4 mmHg (p value <0.001). There was a statistically significant difference between BP in the two groups before and after intervention (p-value <0.001)
Kamal, 2015 [55]	Pakistan, 162	RCT	NM	NM	NM	NM	NM	NM	Intervention group – 1. HBM-based education + Social Cognitive Theory; 2. Usual care; 3. Reminder health information SMS for 2 months. Control: Usual care	Mean SD of Intervention group – DBP: 80 mmHg Control group – DBP: 80.6 mmHg	Mean SD of Intervention group – DBP: 77.9 mmHg; Control group – DBP: 80.5 mmHg. Mean difference Intervention group – DBP: -2.6 mmHg. Control group – DBP: -0.1 mmHg, SBP (p value = 0.678), DBP (p value = 0.06); Not significant

BP measurement in these studies followed standard guidelines. Most of the intervention studies reported pre- and post-intervention systolic BP (SBP) and diastolic BP (DBP) values to identify significant changes. Following the interventions, seven studies reported a significant reduction in blood pressure (p < 0.05) [34-36,47,48,52,55], whereas two studies showed no remarkable decrease [38,45].

Medication Adherence

The relationship between the HBM and medication adherence was examined in 11 studies (Table 2). Among them, three were randomized controlled trials (RCTs) [40,47,55], one was a pre- and post-intervention study [52], and the remaining were cross-sectional [49,50,53-57]. Of these, eight studies were conducted in Asian countries and three in African nations. Four studies implemented HBM-based interventions supplemented with educational sessions, calendar handbooks, SMS reminders, and follow-ups over a duration ranging from 2 to 6 months [40,47,52,55]. Additionally, one study incorporated social cognitive theory with HBM as part of the intervention [55].

**Table 2:** Association of medication adherence with different constructs of HBM or interventions based on them ^a^Adjusted for baseline adherence score, number of pills prescribed daily, dosing frequency, age, gender, employment status, education, use of alarms, missing physician appointments in the previous year, and block design. Study design: CS: cross-sectional, RCT: randomized controlled trial; CRS-HBM: comprehensive reminder system–HBM; +: positively significant; –: negatively significant; MC: missing construct; NM: association not mentioned; NS: not significant; N/A: not applicable.

Reference	Country and Sample Size	Study Design	Association With Different Constructs of HBM						Intervention	Effect of Intervention on Medication Adherence
			Perceived Susceptibility	Perceived Severity	Perceived Benefit	Perceived Barrier	Cues to Action	Self-Efficacy		
Wang, 2020 [47]	China, 174	RCT	NM	NM	NM	NM	MC	NM	Intervention group: Usual care and (CRS-HBM) for 6 months, including: 1. Health belief education; 2. The HBM Calendar Handbook; 3. Weekly short message services; 4. Telephone follow-up interviews. Control group: usual care, including: 1. Health education; 2. Stroke prevention handout; 3. Telephone follow-up by nurses; 4. Follow-up by doctors.	Analyses of variance between groups in change scores from baseline to 3- and 6-month follow-up intervention group: Baseline: 2.96±0.99; 3 months: 3.88±0.24; 6 months: 3.87±0.39. In Control group: Baseline: 3.26±0.86; 3 months: 3.52±0.67; 6 months: 3.34±0.81. For the medication adherence dimension of health behaviors, the time effect, the intervention effect, and the interaction effect of time and group were all statistically significant (p<0.05)
Yazdanpanah, 2019 [40]	Iran, 60	RCT	+	+	+	-	+	+	HBM-based education program; 8 educational sessions; One hour twice a week	Post-test mean score of medication adherence: Intervention group: 6.7±0.5; Control group: 3.7±1.0; Mean score of medication adherence in the intervention group had significantly increased in the post-test phase (P˂0.001) based on the within-group results of the paired t-test.
Allah and Khalil, 2016 [52]	Egypt, 186	Pre- & post- intervention study	+	+	+	-	+	+	Intervention group: HBM-based education program; Control group: Traditional health education on hypertension and compliance to medication and lifestyle regimen	Marked improvement in the HBM group in medication compliance (59.9% to 79.6%) and overall compliance (61.3% to 79.6%); A significant difference was present by comparing pre & post-test results in HBM group (p <0.05).
Kamal, 2015 [55]	Pakistan, 162	RCT	NM	NM	NM	NM	NM	NM	Intervention group: 1. HBM-based education + Social Cognitive Theory; 2. Usual care; 3. Reminder health information SMS for 2 months. Control group: Usual care	Morisky medication adherence score Intervention group: Baseline: 6.6 ± 0.17; 2 months: 7.4 ± 0.93. Control group: Baseline: 6.6 ± 0.16; 2 months: 6.7 ± 1.32; Adjusted difference^a^ (95%CI) 0.54 (0.22–0.85). This difference was found to be statistically significant (p<0.01)
Suhat, 2022 [56]	Indonesia, 180	CS	+	+	NS	-	MC	MC	N/A	N/A
Obirikorang, 2018 [57]	Ghana, 678	CS	+	+	+	-	+	MC	N/A	N/A
Yang, 2016 [49]	China, 745	CS	MC	+	NS	-	NS	+	N/A	N/A
Mahrous, 2015 [53]	Egypt, 135	CS	+	NS	+	-	+	MC	N/A	N/A
Venkatachalam, 2015 [54]	India, 473	CS	+	NS	+	-	+	+	N/A	N/A
Yue, 2015 [50]	China, 232	CS	+	NS	NS	-	+	+	N/A	N/A
Kamran A, 2014 [41]	Iran, 671	CS	+	+	+	-	NM	+	N/A	N/A

Among the HBM variables, perceived barriers had the most frequent construct, showing a significant negative association in nine studies. Perceived susceptibility exhibited a stronger positive association in eight studies. Four interventional studies demonstrated statistically significant improvement in medication adherence, with the remarkable increase in post-intervention mean scores, medication compliance rates, and adherence levels over time [40,47,52,55].

Self-Management

Table 3 shows that 11 studies evaluated the role of the HBM in self-management. Of these, five were cross-sectional [39,42,44,51,58], three were quasi-experimental [43,45,46], two were RCTs [47,48], and the remaining was a nested cohort study [36]. Ten of these studies were conducted in eastern and southwestern Asian countries, and one in South America.

**Table 3:** Association of self-management with different constructs of HBM or interventions based on them *t-test or Mann–Whitney U test between groups, p <0.05. Study design: CS: cross-sectional, QES: quasi-experimental study, RCT: randomized controlled trial; CRS-HBM: comprehensive reminder system–HBM; HPLP Ⅱ total score: health promoting lifestyle profile II; +: positively significant; –: negatively significant; MC: missing construct; NM: association not mentioned; NS: not significant; N/A: not applicable.

Reference	Country and Sample Size	Study Design	Constructs of Health Belief Model on Self-Management						Intervention	Effect of Intervention on Self-Management
			Perceived Susceptibility	Perceived Severity	Perceived Benefit	Perceived Barrier	Cues to Action	Self-Efficacy		
Afshari, 2022 [45]	Iran, 100	QES	+	+	NS	NS	NS	+	HBM-based education program; 3 training sessions in 2 weeks, each lasting one hour	Mean score of self-care behaviors: Intervention group – before intervention: 10.50 ± 2.25; after intervention: 12.02 ± 2.50, p = 0.337. Control group – before intervention: 11.45 ± 2.03; after intervention: 11.36 ± 1.96, p = 0.044. After training, there was an increase in mean scores of self-care in the intervention group with a significant difference between the two groups (p<0.05)
Naeemi, 2022 [43]	Iran, 99	QES	NS	NS	+	NS	+	+	HBM-based educational intervention; three 60-minute training sessions during 3 weeks in 4 groups	Mean score of self-care score: Intervention group – before intervention: 60.02 ± 12.2; 3 months after intervention: 79.4 ± 7.2. Control group: Intervention group – before intervention: 59.1 ± 9.9 3 months; after intervention: 59.7 ± 10. Paired t-test showed that in the experimental group, the mean scores of self-care three months after the intervention were significantly higher than before the intervention (p<0.001)
Wang, 2020 [47]	China, 174	RCT	NM	NM	NM	NM	MC	NM	Intervention group: Usual care and (CRS-HBM) for 6 months, including: 1. Health belief education; 2. The HBM Calendar Handbook; 3. Weekly short message services; 4. Telephone follow-up interviews. Control group: usual care, including: 1. Health education; 2. Stroke prevention handout; 3. Telephone follow-up by nurses; 4. Follow-up by doctors	Total score of health behavior: Intervention group – Baseline: 2.53±0.52; 3 months: 3.16±0.31; 6 months: 3.23±0.35. Control group: Baseline – 2.50±0.42; 3 months: 2.80±0.38; 6 months: 2.75±0.40. A repeated measures analysis of variance showed that the time effect, the intervention effect, and the interaction effect of time and group were all statistically significant (p<0.001)
Zhang, 2020 [36]	China, 174	Nested Cohort	NM	NM	NM	NM	MC	NM	(CRS-HBM) for 6 months, including: 1. Health belief education; 2. The HBM Calendar Handbook; 3. Weekly short message services; 4. Telephone follow-up interviews	HPLP II total score intervention group: Baseline – 2.55 ± 0.51; 6 months: 3.23±0.35. Control group: Baseline – 2.54 ± 0.44; 6 months: 2.75 ± 0.40. Six months after discharge, both groups showed improvement in health behaviors, and when the groups were compared in their total HPLP II score, the intervention group showed statistically significant improvement over the control group. Direct positive effect (β = .391, p < .001); Indirect positive effects (β = .186, p = .002)
Wan, 2018 [48]	China, 174	RCT	NM	NM	NM	NM	NM	NM	Intervention group: Usual stroke education and (CRS-HBM) for 6 months, including: 1. Health belief education; 2. The HBM Calendar Handbook; 3. Weekly short message services; 4. Telephone follow-up interviews. Control group: Usual stroke education	Total score of health behavior over time and between groups: Intervention group: Baseline: 2.54 ± 0.52 3 months: 3.16 ± 0.32 Changes: 0.61±0.41*. Control group: Baseline – 2.51 ± 0.42; 3 months: 2.79 ± 0.38; Changes: 0.28 ± 0.42*. Three months post-discharge, both groups showed improved health behaviors, yet when the groups were compared in their total HPLP II score, the difference was statistically significantly higher for the intervention group (P < 0.001)
Khorsandi, 2017 [46]	Iran, 91	QES	+	+	+	-	NS	NS	HBM-based education program	p values of changes in mean score after the intervention of different constructs: Susceptibility – 0.041; Awareness – 0.013; Benefit – 0.001; Barrier – 0.001; Practice – 0.001; Self-efficacy: 0.010; Action: 0.001
Sadeghi, 2022 [42]	Iran, 200	CS	+	NS	+	-	NS	+	N/A	N/A
Zareban, 2022 [44]	Iran, 527	CS	NS	MC	+	-	NS	+	N/A	N/A
Larki, 2021 [39]	Iran, 152	CS	+	+	NS	NS	MC	+	N/A	N/A
Ma, 2018 [51]	China, 382	CS	+	+	+	-	MC	+	N/A	N/A
Barros, 2014 [58]	Brazil, 133	CS	+	+	NS	NS	MC	MC	N/A	N/A

HBM-based interventions such as training sessions, educational sessions, lectures, educational films, group discussions, educational pamphlets, and educational intervention programs were used in six studies, with durations ranging from two weeks to six months [36,43,45-48]. These studies showed statistically significant increases in self-management, health behavior, and self-care scores after the interventions (p < 0.05). Among the HBM components, perceived susceptibility [39,42,45,46,51,58] and self-efficacy [39,42-45,51] strongly influenced self-management in six studies.

Discussion

This review aimed to investigate the role of the HBM in hypertension. By assessing its role in various aspects of hypertension management, the review identified significant associations between the HBM and outcomes such as blood pressure reduction, medication adherence, and self-management.

Among the 24 articles included in this study, only 13 employed all six constructs of the HBM. The construct most frequently omitted was “cue to action,” which has been identified as the least utilized construct in several other studies [24]. Three of the studies implemented multifaceted interventions that included calendar handbooks, weekly short messages, telephone follow-ups, and other methods in addition to HBM education sessions [36,47,48]. Our study did not examine whether interventions using multifaceted components were more effective than those using a single component, although one systematic review revealed no difference in effectiveness between the two [59]. Only one study utilized more than one behavioral theory as a framework for intervention [55]. Although incorporating multiple behavioral change theories could enhance effectiveness, this study did not explore that approach [60].

The HBM serves as an effective intervention framework for health education and has been delivered through methods such as workshops, digital reminder systems, or face-to-face lessons, resulting in a notable reduction in blood pressure across numerous studies [34-36,47,48,52]. One study reported no significant reduction in blood pressure; however, the absence of a control group might limit the comparability of the results [38]. Additionally, the shorter intervention period and follow-up assessment in one study may help explain the lack of significant results in terms of blood pressure reduction [45]. Although not statistically significant, a reduction in diastolic blood pressure was observed in a study by Kamal and colleagues [55]. They also incorporated social cognitive theory alongside the HBM, so it is unclear whether using the HBM alone would have led to different outcomes.

In this review, we found that interventions based on the HBM can significantly help individuals with hypertension adhere to medication [40,47,52,55]. Our findings are similar to those of another review in which most of the included studies reported significant outcomes [33]. Adherence is adversely affected by perceived barriers [61-63]. Our review revealed that barriers were consistently and significantly associated in all studies, followed by perceived susceptibility. Previous reviews also reported these factors as the most common constructs for medication adherence, although they found perceived benefit to be another predominant construct [33]. Unlike the abovementioned review, this study did not measure the effect size of the outcomes, which may have limited our ability to detect similar findings. Perceived barriers have also been found to exert the most significant negative influence on adherence in other chronic conditions [61-63].

Among the 11 studies reviewed, six employed the HBM as an intervention and demonstrated significant improvements in self-management health behaviors [36,43,45-48]. Previous research has also highlighted the effectiveness of HBM-based interventions in promoting self-care behaviors [64,65]. Additionally, a prior review identified positive associations between HBM constructs and health behaviors [32]. In our review, the constructs most significantly associated with self-care were perceived susceptibility and self-efficacy [39,42-46,51,57]. Our findings align with previous research where these constructs were found to be the most significant [66-68].

Unlike previous reviews, this study provided a detailed analysis of blood pressure reduction effectiveness and incorporated medication adherence and self-management, which are closely related to successful BP control. However, this review also raises several issues that require further investigation. In most of the studies, the intervention duration ranged from as short as two weeks to up to six months. This wide variation in duration warrants future research to determine the minimum effective time for interventions. Additionally, it remains unclear how long the effects of these interventions last after discontinuation. Previous studies suggest that the positive impacts of such interventions can persist for up to five years, alleviating concerns about long-term consequences [69]. A longitudinal analysis of outcomes from these studies could provide clearer insights into this matter. A comparison of studies involving different behavioral theory-based interventions should also be conducted to determine which intervention yields the best results.

This study has several limitations that are important to acknowledge. Studies in languages other than English were excluded, potentially introducing language bias. The use of constructs across studies was heterogeneous, with some incorporating all constructs and others omitting one or two. However, the researchers did not provide explanations for why certain constructs were excluded. Another limitation was that the study did not measure the effect size of the outcome variables, hindering a comprehensive evaluation of results. Due to heterogeneity of results and lack of effect size measurement, we missed the opportunity for a further meta-analysis. Modifying factors related to blood pressure management were not discussed. Many studies did not address the health literacy or education level of participants, which could impact their ability to understand the various constructs involved. Additionally, the HBM may be less effective in different sociocultural contexts if it is not culturally adapted.

## Conclusions

The HBM has the potential to predict health behaviors among individuals with hypertension. Interventions grounded in this model hold promise for effective hypertension management. The extensive evidence gathered from these studies may inform policy initiatives aimed at promoting a more proactive approach to cardiovascular health at the societal level, in addition to promoting patient-centered care in clinical practice.

## Appendices

**Table 4:** JBI checklist Risk of bias for cross-sectional studies.

Author, Year	Q1. Were the criteria for inclusion in the sample clearly defined?	Q2. Were the study subjects and the setting described in detail?	Q3. Was the exposure measured in a valid and reliable way?	Q4. Were objective, standard criteria used for measurement of the condition?	Q5. Were confounding factors identified?	Q6. Were strategies to deal with confounding factors stated?	Q7. Were the outcomes measured in a valid and reliable way?	Q8. Was appropriate statistical analysis used?	Total Score	Quality Assessment
Suhat et al., 2022 [56]	Yes	Yes	Yes	Yes	No	No	Yes	Yes	6	Moderate
Sadeghi et al., 2022 [42]	Yes	Yes	Yes	Yes	No	No	Yes	Yes	6	Moderate
Zareban et al., 2022 [44]	Yes	Yes	Yes	Yes	No	No	Yes	Yes	6	Moderate
Larki, Reisi and Tahmasebi, 2021 [39]	Yes	Yes	Yes	Yes	No	No	Yes	Yes	6	Moderate
Obirikorang et al., 2018 [57]	Yes	Yes	Yes	Yes	No	No	Yes	Yes	6	Moderate
Ma, 2018 [51]	Yes	Yes	Yes	Yes	No	No	Yes	Yes	6	Moderate
Yang et al., 2016 [49]	Yes	Yes	Yes	Yes	No	No	Yes	Yes	6	Moderate
Mahrous, 2015 [53]	Yes	Yes	Yes	Yes	No	No	Yes	Yes	6	Moderate
Venkatachalam et al., 2015 [54]	Yes	Yes	Yes	Yes	No	No	Yes	Yes	6	Moderate
Yue et al., 2015 [50]	Yes	Yes	Yes	Yes	Yes	Yes	Yes	Yes	8	Low
Kamran et al., 2014 [41]	Yes	Yes	Yes	Yes	No	No	Yes	Yes	6	Moderate
Alves Barros et al., 2014 [58]	Yes	Yes	Unclear	Yes	No	No	Yes	Yes	5	Moderate
